# Reference intervals for complete blood count from Umbilical Cord Blood in newborns and comparison with Venous Blood Values

**DOI:** 10.12669/pjms.37.2.2526

**Published:** 2021

**Authors:** Mehmet Gunduz, Hayrettin Temel

**Affiliations:** 1 Dr. Mehmet Gunduz, M.D. Assistant Professor, Istanbul Medipol University School of Medicine, Department of Pediatrics, Medipol Sefaköy Hospital, Sefaköy, Istanbul, Turkey; 2 Dr. Hayrettin Temel, M.D. Assistant Professor, Istanbul Medipol University School of Medicine, Department of Pediatrics, Medipol Mega Hospital Complex, Bağcılar, Istanbul, Turkey

**Keywords:** Complete Blood Count, Reference range, Umbilical cord

## Abstract

**Background and Objective:**

Umbilical cord blood which can be obtained by a non-invasive method can be informative about the clinical status of the newborn. It was aimed to establish reference intervals for umbilical cord blood parameters, and to compare complete blood count results between umbilical cord and venous blood samples in this study.

**Methods:**

This study was conducted at Medipol University Sefaköy Hospital, Department of Pediatrics, Istanbul, Turkey. A total of 1898 newborns who were born in a two-year period between January 2018 and December 2019 were included in the study. Venous blood samples were taken from 184 of them, and umbilical cord blood samples were taken from 1714 newborns.

**Results:**

The percentiles were determined according to gender and delivery method for the hematological parameters of umbilical cord blood. While mean platelet, eosinophil and mean corpuscular volume values were similar between the groups (p>0.05 for each), and significant differences were found between the groups in terms of all other mean hematological parameters (p<0.05 for each).

**Conclusion:**

The results of the complete blood count of umbilical cord blood samples can provide reliable information about the newborn. There are significant differences between umbilical cord and venous blood samples in terms of hematological parameters. For these reasons, it is necessary to determine reliable value ranges for umbilical cord blood hematological parameters in newborns. Data of our study can be a guide for further studies and clinicians.

## INTRODUCTION

Complete blood count (CBC) is an important test that provides reliable basic information about the clinical condition of the patient quickly. The information giving clues as to whether the patient is infected or even that the infection is bacterial or viral and that it is acute or chronic can be obtained easily. In addition, findings on some disorders such as anemia, thrombocytopenia, thrombocytosis and neutropenia and life-threatening diseases such as leukemia can be provided. CBC is often used to monitor the course of the disease or treatment as well as the diagnosis.^1^-^3^

CBC provides the first blood results to show the health status of the newborn. However, this test has some negative aspects, especially in newborns, such as being a minimally invasive procedure, difficulty in finding the vein to be collected, not enough blood can be taken for the test and some possible local complications. For these reasons, some studies have been conducted on whether or not to use umbilical cord blood instead of venous blood for CBC in newborns.^4^,^5^

There is no risk of complications since umbilical cord blood is taken by a non-invasive method for the newborn. However, it is still being investigated whether the CBC with umbilical cord blood can provide reliable information as much as venous blood. The reference intervals have been established for hematological parameters in the CBC of umbilical cord blood in some countries.^6^-^9^ We know that the reference intervals for hematological parameters for umbilical cord blood have not been determined for Turkey yet. In this present study, it was aimed to establish reference intervals for umbilical cord blood parameters, and to compare CBC results between umbilical cord blood and venous blood.

## METHODS

This study was conducted at Medipol University Sefaköy Hospital, Department of Pediatrics, Istanbul, Turkey. This study was approved by the local Ethics Committee/IRB with 10840098-604.01.01-E.12268 and March 11, 2020 and was conducted retrospective. Written and signed informed consent forms were obtained from the mothers for all samples included in the study.

### Patients and Inclusion Criteria

A total of 1898 newborns who were born in our gynecology and obstetrics clinic and admitted to the neonatal unit in the two-year period between January 2018 and December 2019 were included in the study. Venous blood samples were taken from 184 of them, and umbilical cord blood samples were taken from 1,714 newborns.

Babies born after 36 weeks of gestation were included in the study. Those who were born by both cesarean and vaginal ways were accepted to the study. Those who were stillborn, preterms, those admitted to the intensive care unit and those with Rh or ABO incompatibility were excluded. Also, mothers with malignant tumors or fever higher than 38°C, premature membrane rupture older than 24 hours, maternal perinatal complications, newborns with congenital anomalies, placental abruption and delicate or very short umbilical cord deliveries were excluded.

### Blood Samples and Analysis of Tests

Umbilical cord blood samples were taken after the baby was born, after the umbilical cord was clamped before the placenta was removed or after the delivery process was completely completed. The sampling was done by the clinician who delivered from the plasenta side. Venous blood samples were routinely taken. Blood samples taken in total 0.5 ml were transferred to EDTA containers (Becton Dickinson, Franklin Lanes, NJ, USA). CBC tests were performed on Beckman Coulter AcT diff2 (Brea, CA, USA) automated analyzer.

### Statistical analysis

Statistical analyzes were performed using SPSS 25.0 (SPSS Inc., Chicago, IL, USA) software. Whether the distribution was normal for numerical variables was checked by Kolmogorov-Smirnov test, and it was confirmed that all variables were normally distributed. The reference intervals for blood values were calculated to be between 2.5% and 97.5%.^7^,^8^ Descriptive statistics for continuous variables are given as mean ± standard deviation. The mean differences for genders, mode of delivery, and each blood value between the umbilical cord and venous blood were analyzed with the Independent Samples T-Test. p<0.05 values were considered statistically significant. Bonferroni correction was made where appropriate.

## RESULTS

A total of 974 (51.3%) of the newborns were boys and 924 (48.7%) were girls. The groups were similar according to gender distribution (p=0.707). A total of 715 (37.7%) cases were born by normal vaginal delivery, and 1,183 (62.3%) newborns were delivered by cesarean method. The rate of cases born by cesarean was significantly higher in the umbilical cord group (p=0.008).

The distribution of blood values in 2.5% and 97.5% percentile ranges and mean ± standard deviation values and comparisons by genders are shown in Table-I and II. The mean hematocrit, mean corpuscular volume (MCH), lymphocyte, monocyte and mean platelet volume (MPV) values and basophil percentage were similar between genders (p> 0.05 for each), and all other mean blood values were significantly higher in male newborns than female ones (p <0.05 for each).

**Table-I:** Mean ± standard deviation and range (2.5-97.5%) values of complete blood count tests of umbilical cord vein.

	*Mean ± SD*				*Range (2.5-97.5%)*		

	*Total*	*Male*	*Female*	*p*	*Total*	*Male*	*Female*
RBC (10^6^/mL)	4.4 ± 0.5	4.4 ± 0.6	4.3 ± 0.5	0.003	3.44-5.55	3.43-5.73	3.44-5.4
Hb (g/dL)	15.6 ± 1.9	15.7 ± 1.9	15.4±1.7	0.002	12.4-19.9	12.5-20.4	12.3-19.5
Hct (%)	45.1 ± 5.2	45.3 ± 5.4	44.9±5	0.083	36-56.4	36.1-57.6	35.9-55.3
MCV (mm^3^)	103.3 ± 4.6	103 ± 4.7	103.7±4.4	0.001	93.8-112.1	93-111.8	94-112.4
MCH (pg/cell)	35.7 ± 1.9	35.7 ± 2	35.7±1.7	0.547	31.8-39.2	31.7-39.4	31.8-38.9
MCHC (Hb/cell%)	34.5 ± 1.2	34.7 ± 1.3	34.4±1	<0.001	33-36.5	33-36.7	33-36.2
Platelets (10^3^/mL)	250.1 ± 57.6	244.5 ± 55.9	256.1±58.9	<0.001	128-362	129-353	128-367
WBC (10^3^/mL)	13.7 ± 4.2	13.2 ± 4.2	14.2±4.2	<0.001	7.35-23.42	7.02-23	8.29-23.6

**RBC:** Red blood cells, **Hb:** Hemoglobin, **Hct:** Hematocrit, **MCV:** Mean corpuscular volume, **MCH:** Mean corpuscular hemoglobin, **MCHC:** Mean corpuscular hemoglobin concentration, **WBC:** White blood cells, **SD:** Standard deviation.

**Table-II:** Mean ± standard deviation and range (2.5-97.5%) values of differential count of white blood cells of umbilical cord vein.

	*Mean ± SD*			*p*	*Range (2.5-97.5%)*		

	*Total*	*Male*	*Female*		*Total*	*Male*	*Female*
Total neutrophils (10^9^/L)	6.8 ± 3	6.4 ± 2.8	7.3 ± 3	<0.001	2.63-13.6	2.27-13.3	2.89-13.7
Lymphocytes (10^9^/L)	4.8 ± 1.5	4.8 ± 1.4	4.9 ± 1.5	0.27	2.77-8.37	2.69-8.19	2.8-8.49
Monocytes (10^9^/L)	1.5 ± 0.5	1.4 ± 0.5	1.5 ± 0.5	0.076	0.68-2.63	0.61-2.65	0.75-2.6
Eozinophils (10^9^/L)	0.5 ± 0.3	0.5 ± 0.3	0.4 ± 0.3	0.017	0.09-1.16	0.08-1.24	0.1-1.07
Basophils (10^9^/L)	0.1 ± 0.1	0.1 ± 0.1	0.1 ± 0.1	0.034	0.02-0.43	0.02-0.4	0.02-0.5
Neutrophils (%)	48.6 ± 9	47.1 ± 8.8	50.1 ± 8.9	<0.001	29.7-65.6	28.6-64.4	30.5-66.2
Lymphocytes (%)	36.5 ± 8.6	37.6 ± 8.5	35.4 ± 8.6	<0.001	20.3-55	20.5-56.6	19.8-53.6
Monocytes (%)	10.7 ± 2.1	10.8 ± 2.2	10.5 ± 2	<0.001	6.9-15.1	6.8-15.2	7-14.5
Eozinophils (%)	3.5 ± 2	3.7 ± 2.1	3.2 ± 1.8	<0.001	0.7-8.6	0.7-9.1	0.7-7.8
Basophils (%)	0.8 ± 0.6	0.8 ± 0.6	0.8 ± 0.6	0.689	0.2-2.4	0.2-2.4	0.2-2.5
MPV (fL)	9.6 ± 0.7	9.6 ± 0.7	9.7 ± 0.7	0.284	8.5-11.1	8.5-11.1	8.5-11.1

**MPV:** Mean platelet volume, **SD:** Standard deviation.

The results of the samples taken from umbilical cord and venous blood samples are shown in Table-III. Accordingly, while the mean MCH, platelet and eosinophil values were similar between the groups (p>0.05 for each), significant differences were found between the groups in terms of all other mean blood values (p<0.05 for each). Comparison of mean hematological values and reference value intervals for umbilical blood samples by delivery method are shown in Table-IV.

**Table-III:** Comparison between umbilical cord versus venous blood samples.

	*Total*			*Male*			*Female*		

	*Umbilical cord*	*Venous*	*p*	*Umbilical cord*	*Venous*	*p*	*Umbilical cord*	*Venous*	*p*
RBC(10^6^/mL)	5±0.6	4.4±0.5	<0.001	5 ± 0.6	4.4±0.6	<0.001	5.1±0.6	4.3±0.5	<0.001
Hb(g/dL)	17.8±2.2	15.6±1.9	<0.001	17.7 ± 2	15.7±1.9	<0.001	17.9±2.4	15.4±1.7	<0.001
Hct(%)	51.1±6	45.1±5.2	<0.001	50.8 ± 5.7	45.3±5.4	<0.001	51.4±6.2	44.9±5	<0.001
MCV(mm^3^)	102±4.6	103.3±4.6	<0.001	102±4.6	103±4.7	0.056	102.1±4.7	103.7±4.4	0.056
MCH(pg/cell)	35.6±2	35.7±1.9	0.383	35.7±2	35.7±2	0.815	35.5± .9	35.7±1.7	0.815
MCHC(% Hb/cell)	34.9±1.3	34.5±1.2	<0.001	35±1.5	34.7±1.3	0.044	34.7±1	34.4±1	0.044
Platelets(10^3^/mL)	244.2±61.3	250.1±57.6	0.187	242.9±60.2	244.±55.9	0.803	245.4±62.7	256.1±58.9	0.803
WBC(10^3^/mL)	17.2±5	13.7±4.2	<0.001	16.6±5.3	13.2±4.2	<0.001	17.8±4.6	14.2±4.2	<0.001
Total neutrophils (10^9^/L)	8.3±3.6	6.8±3	<0.001	7.7±3.6	6.4±2.8	<0.001	8.9±3.5	7.3±3	<0.001
Lymphocytes (10^9^/L)	6.5±2.1	4.8±1.5	<0.001	6.7±2.3	4.8±1.4	<0.001	6.4±1.9	4.9±1.5	<0.001
Monocytes (10^9^/L)	1.7±0.6	1.5±0.5	<0.001	1.7±0.7	1.4±0.5	<0.001	1.8±0.6	1.5±0.5	<0.001
Eozinophils (10^9^/L)	0.5±0.3	0.5±0.3	0.223	0.5±0.3	0.5±0.3	0.783	0.5±0.3	0.4±0.3	0.783
Eozinophils (10^9^/L)	0.2±0.1	0.1±0.1	<0.001	0.2±0.1	0.1±0.1	<0.001	0.2±0.1	0.1±0.1	<0.001
Basophils (10^9^/L)	47±10.2	48.6±9	0.028	44.7±10.5	47.1±8.8	0.014	49.4±9.4	50.1±8.9	0.014
Neutrophils(%)	39.1±10.1	36.5±8.6	<0.001	41.4±10.7	37.6±8.5	<0.001	36.9±9	35.4±8.6	<0.001
Lymphocytes (%)	10.1±2.3	10.7 ± 2.1	<0.001	10.1±2.5	10.8±2.2	0.001	10±2.2	10.5± 2	0.001
Monocytes (%)	2.9±1.5	3.5±2	<0.001	3±1.5	3.7±2.1	0.002	2.8±1.5	3.2±1.8	0.002
Eozinophils (%)	0.9±0.6	0.8±0.6	0.01	0.9±0.6	0.8±0.6	0.095	0.9±0.5	0.8±0.6	0.095
Basophils (%)	9.8±0.7	9.6±0.7	0.001	9.7±0.7	9.6±0.7	0.673	10±0.7	9.7±0.7	0.673
MPV(fL)	±0.6	4.4±0.5	<0.001	5±0.6	4.4±0.6	<0.001	5.1±0.6	4.3±0.5	<0.001

**RBC:** Red blood cells, **Hb:** Hemoglobin, **Hct:** Hematocrit, **MCV:** Mean corpuscular volume, **MCH:** Mean corpuscular hemoglobin, **MCHC:** Mean corpuscular hemoglobin concentration, **WBC:** White blood cells, **MPV:** Mean platelet volume, **SD:** Standard deviation.

**Table-IV:** Comparison between normal spontaneous vaginal delivery and cesarean birth.

	*Mean ± SD*			*Range (2.5-97.5%)*	

	*Normal*	*Cesarean*	*p*	*Normal*	*Cesarean*
RBC (10^6^/mL)	4.5±0.5	4.3±0.5	<0.001	3.69-5.58	3.36-5.54
Hb (g/dL)	16.1±1.7	15.3±1.9	<0.001	13.2-19.7	12.2-20
Hct (%)	46.6±4.7	44.3±5.3	<0.001	38.1-56.3	35.5-56.7
MCV (mm^3^)	102.8± 4.6	103.6±4.5	<0.001	93-111.1	94.2-112.9
MCH (pg/cell)	35.5±1.9	35.8±1.9	0.001	31.5-38.8	31.9-39.4
MCHC (% Hb/cell)	34.5 ± 1.2	34.5±1.2	0.801	33-36.6	33.1-36.5
Platelets (10^3^/mL)	254.4 ± 57.8	247.6±57.4	0.019	145-366	124-357
WBC (10^3^/mL)	14.8±4.2	13±4.1	<0.001	8.14-23.65	7.02-23.36
Total neutrophils (10^9^/L)	7.5±3	6.4±2.8	<0.001	3.19-14.2	2.25-13.34
Lymphocytes (10^9^/L)	5.2±1.6	4.6±1.4	<0.001	2.8-8.79	2.75-8.14
Monocytes (10^9^/L)	1.5±0.5	1.4±0.5	<0.001	0.76-2.71	0.65-2.62
Eozinophils (10^9^/L)	0.5±0.3	0. ±0.3	0.824	0.1-1.1	0.08-1.16
Basophils (10^9^/L)	0.1±0.1	0.1±0.1	<0.001	0.02-0.54	0.02-0.35
Neutrophils (%)	49.7±8.5	47.9±9.2	<0.001	32.5-66.6	28.1-64.6
Lymphocytes (%)	35.8±8.2	36.9±8.9	0.010	20.1-52.8	20.5-56.9
Monocytes (%)	10.4±2	10.8±2.2	<0.001	7-14.6	6.9-15.2
Eozinophils (%)	3.2±1.9	3.6±2.1	<0.001	0.8-8.1	0.7-8.8
Basophils (%)	0.9±0.7	0.8±0.5	<0.001	0.2-2.6	0.2-2.3
MPV (fL)	9.6±0.7	9.6±0.7	0.778	8.4-10.9	8.5-11.1

**RBC:** Red blood cells, **Hb:** Hemoglobin, **Hct:** Hematocrit, **MCV:** Mean corpuscular volume, **MCH:** Mean corpuscular hemoglobin, **MCHC:** Mean corpuscular hemoglobin concentration, **WBC:** White blood cells, **MPV:** Mean platelet volume, **SD:** Standard deviation.

## DISCUSSION

Some studies have been conducted as to whether the umbilical cord blood can be an alternative for venous blood. It has been reported that blood taken from the umbilical cord provides information to the clinician about various perinatal problems such as fetal hematopoiesis, infection and chorioamnionitis, perinatal asphyxia, and meconium inhalation.^6^,^7^,^10^ Although umbilical cord blood can provide important information, reliable reference value intervals for umbilical cord blood test results have not been determined, except a few studies. In addition, there are also few reports comparing umbilical cord and venous blood results.^4^,^6^-^8^ In our study, reference intervals were determined for hematological parameters with CBC results from umbilical cord blood, and comparisons were made with venous blood results. In this way, it is examined whether it is necessary to determine the reference intervals for umbilical cord blood.

It was reported that the mean values in the CBC made from umbilical cord blood samples varied regionally or racially.^6^,^7^,^10^ Therefore, it is an appropriate approach to determine reference values that can be a guide for our country. In our study, reference values were determined for the umbilical cord blood hematological parameters for our country according to both genders and delivery methods.

In the studies in which umbilical cord and venous blood samples taken simultaneously from newborns, a significant difference was reported between these two samples in terms of CBC results.^6^,^11^-^13^ In a study conducted with 174 newborns, it was found that leukocyte, neutrophil and lymphocyte counts were significantly higher in umbilical cord blood.^6^ However, the authors found the groups similar in terms of mean monocyte, eosinophil and platelet counts. In their correlation analysis, they found that leukocyte and hemoglobin values correlated significantly between umbilical cord and venous blood. These researchers stated that determining reference intervals for umbilical cord blood, which is easy to take, will provide greater convenience.^6^ In another study, it was reported that the results of CBC from the umbilical cord and venous blood samples showed high correlation.^14^ Also, it was found that leukocyte, neutrophil, lymphocyte, eosinophil, mean corpuscular hemoglobin (MCH), MCV and MPV values showed high correlation between the two blood samples in another study. They reported that other hematological parameters were correlated, albeit low.^15^-^17^ These researchers stated that these blood samples can be taken from newborns with reference value intervals for umbilical cord blood, and emphasized that if abnormal values are seen, it can be diagnosed with a more invasive procedure, venous blood sampling.^14^-^20^

In our study, unlike these studies, blood samples were taken from two different groups. However, the reliability of the results was increased by including almost two thousand newborns in our study. We found that most hematological parameters differ significantly between the umbilical cord and venous blood samples. All these findings show that umbilical cord blood values may increase and decrease in accordance with venous blood values, but umbilical cord and venous samples are different in terms of mean values of hematological parameters. According to these findings, umbilical cord blood can give reliable results about the clinical condition of the newborn as much as venous blood sample, but it is not possible to interpret the umbilical cord blood results according to venous blood reference values. In this context, reliable reference values should be determined for hematological parameters in the CBC made from umbilical cord blood.

Chang et al.^7^ determined reference intervals for umbilical cord blood hematological parameters in their large study. In addition, they reported that there were significant differences between normal spontaneous vaginal delivery and cesarean delivery in terms of mean hematological values. For this reason, they have determined separate reference values for spontaneous vaginal delivery and cesarean delivery. In our study, significant differences were found between spontaneous vaginal delivery and cesarean delivery in terms of mean hematological parameter values. Therefore, separate umbilical cord reference intervals were determined for both delivery methods.

### Limitations of the study

The cases included in the study were newborns who were born in only one hospital. Therefore, increasing the reliability of the reference values by conducting multi-center studies will be a suitable approach. In the present study, two different blood samples were not taken from newborns. Therefore, the relationship between blood samples belonging to the same newborn could not be evaluated. However, the results of the analysis have been strengthened by keeping the umbilical cord and venous blood groups much wider than other studies.

## CONCLUSION

According to the findings of our study, the results of CBC, which can be obtained from umbilical cord blood samples that can be obtained easily and non-invasively from newborns, can provide reliable information about the newborn. The results obtained in our study show that there are significant differences between umbilical cord blood and venous blood in terms of blood parameters. For these reasons, it is necessary to determine the high reliability value ranges for umbilical cord blood hematological parameters in newborns. Data of our study can be a guide for further studies and clinicians.

### Authors’ Contribution:

**MG:** Planning, performing, analyzing data, preparing the manuscript and is responsible for integrity of the study.

**HT:** Preparing the manuscript.
